# Gut microecology in critical illness: mechanisms, biomarkers, and therapeutic strategies — a critical appraisal

**DOI:** 10.3389/fgstr.2026.1902608

**Published:** 2026-08-05

**Authors:** Liang Tian, Shanshan Xu, Xueping Xie, Man Wang, Lina Huang

**Affiliations:** 1 Department of Pharmacy, Shanghai Deji Hospital, Qingdao University, Shanghai, China; 2 Department of Intestinal Microecology, Shanghai Deji Hospital, Qingdao University, Shanghai, China; 3 Intensive Care Unit, Shanghai Deji Hospital, Qingdao University, Shanghai, China

**Keywords:** critical illness, dysbiosis, ecological principles, ecosystem collapse, GRADE evidence, gut microbiome, precision microbiome medicine, short-chain fatty acids

## Abstract

Critical illness is associated with a predictable ecological collapse of the gut microbiome. Within 48 hours of ICU admission, three convergent selective pressures—luminal oxygen enrichment from mucosal inflammation, nitrate provision by iNOS, and antibiotic-mediated niche clearance—drive a phase transition from obligate-anaerobe-dominated communities to pathogen-dominated monocultures, compromising barrier integrity and immune homeostasis. Multi-omics integration recasts this as a cross-kingdom phenomenon encompassing fungi, bacteriophages, and the metabolite networks linking them to host immunity. Whether this collapse directly causes organ dysfunction or merely marks disease severity remains the central unresolved question. This critical review examines evidence across six domains: the healthy microbiota as an ecological benchmark; patterns and temporal dynamics of ICU dysbiosis; molecular mechanisms across four gut–organ axes; clinical consequences including sepsis, nosocomial infection, acute gastrointestinal injury, and multiple organ dysfunction syndrome (MODS); biomarkers and machine-learning predictive models; and therapeutic strategies evaluated using the Grading of Recommendations Assessment, Development and Evaluation (GRADE) framework. Only early enteral nutrition (GRADE: HIGH) and antimicrobial stewardship (GRADE: MODERATE–HIGH) have Phase III evidence supporting routine ICU use. The repeated failures of probiotic trials reflect design limitations—lack of patient stratification, strain mismatch, and soft endpoints—though biological efficacy limitations cannot be excluded. We conclude that ICU dysbiosis is best understood as ecosystem collapse driven by convergent selective pressures, not random taxonomic disturbance. Therapeutic strategies should target functional outputs—SCFAs, bile acids, indole derivatives—rather than species composition. The resilient minority (15–20% of patients who maintain diversity) holds keys to prevention science. A clinically actionable platform requires three components: rapid ecological risk assessment, endotype classification, and mortality-powered randomized controlled trials targeting functional restoration.

## 1 Introduction

Critical illness continues to be a major cause of death worldwide, even with progress in hemodynamic resuscitation, ventilatory support, and antimicrobial therapy (1, 2). Recent scientific interest has concentrated on a shared pathophysiological link among sepsis, acute respiratory distress syndrome (ARDS), and multiple organ dysfunction syndrome (MODS): the gut. In the 1980s, Carrico, Meakins, and Marshall proposed the gastrointestinal tract as the ‘motor’ of MODS, a concept developed before the advent of culture-independent microbiology (3, 4). This hypothesis has since been bolstered by insights from the sequencing revolution.

**Figure 1:**
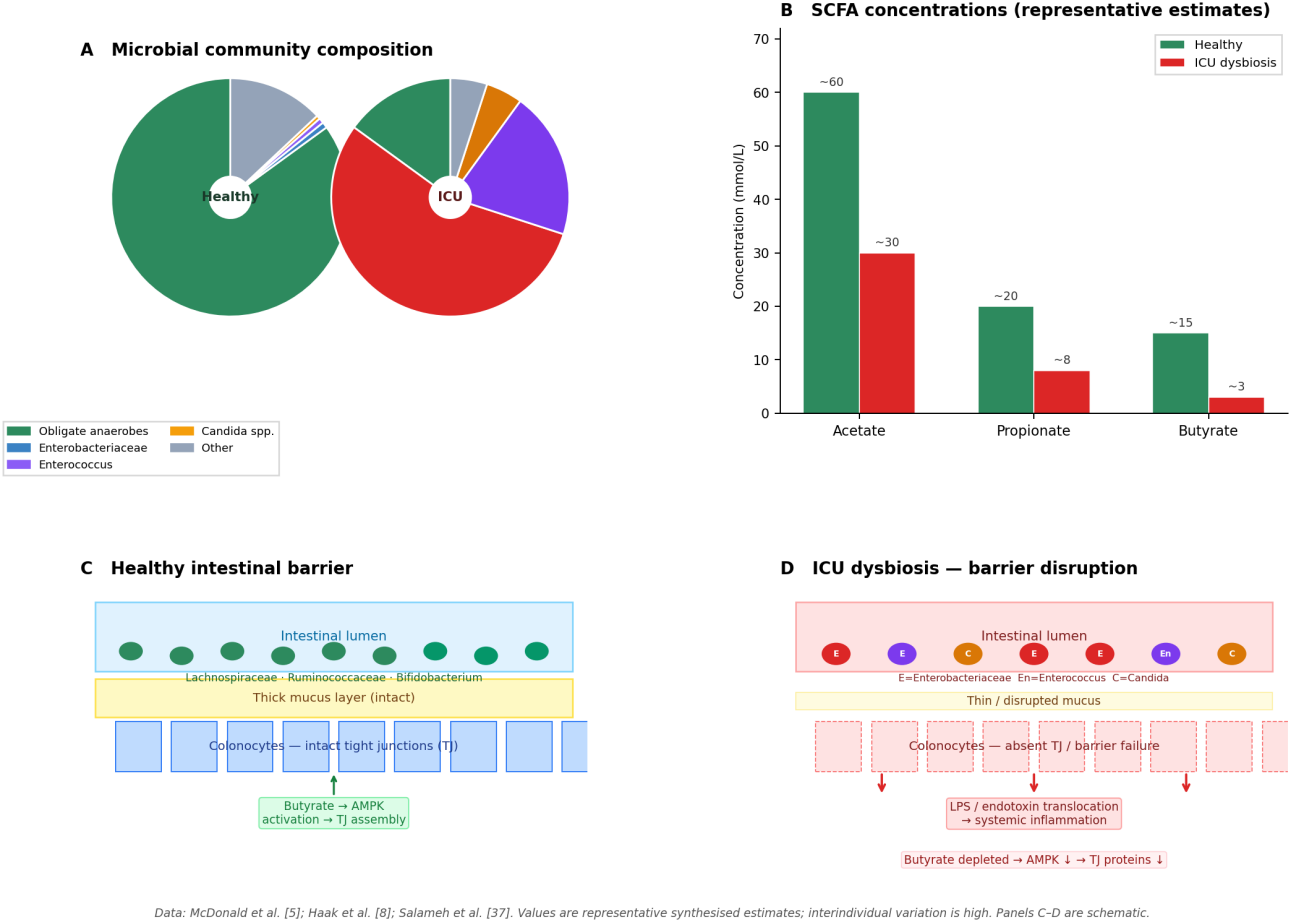
Healthy versus ICU gut microecology. **(A)** Microbial community composition: healthy—obligate anaerobes ~85%, Enterobacteriaceae and Enterococcus each <1%; ICU dysbiosis—Enterobacteriaceae ~55%, Enterococcus ~20%, *Candida* spp. ~5%, obligate anaerobes ~15%. **(B)** Representative SCFA concentrations: acetate ~60 vs ~30 mmol/L; propionate ~20 vs ~8 mmol/L; butyrate ~15 vs ~3 mmol/L. **(C)** Healthy barrier: intact tight junctions (TJ), thick mucus, abundant butyrate from Lachnospiraceae/Ruminococcaceae, AMPK-driven TJ assembly. **(D)** ICU dysbiosis: thin mucus, absent TJ, pathogen dominance, LPS/endotoxin translocation. Values are representative synthesized estimates [McDonald et al. (5); Haak et al. (8); Salameh et al. (9)]; interindividual variation is high. Panels **(C–D)** are schematic. TJ, tight junction; AMPK, AMP-activated protein kinase; LPS, lipopolysaccharide.

Culture-independent techniques have revealed a highly complex gut ecosystem. Disruption of this ecosystem during critical illness is reproducible and consistently linked to the worst outcomes (5, 7, 10) (**Figure 1**; **Table 1**). Metabolomics, mycobiomics, and viromics have demonstrated that ICU dysbiosis extends beyond bacteria, indicating a cross-kingdom ecosystem collapse that includes fungi, bacteriophages, and the metabolite networks connecting them to host immune function (8). Multi-Omics Factor Analysis (MOFA) now extracts latent biological factors that capture microbial, immunological, and metabolic covariation simultaneously, offering a richer substrate for risk prediction than any single analyte (11).

**Table 1:** Key ICU microbiome cohort studies.

Study (year)	Design	N	Method	Key findings	Outcome association
McDonald et al. (5) 2016	Cross-sectional + longitudinal	162 ICU + 199 controls	16S V4	Diversity collapse within 48 h; Bifidobacterium/Lachnospiraceae depleted; Enterobacteriaceae/Enterococcus expanded; ~20% maintain Shannon >3.0	Diversity loss correlated with longer ICU stay and mortality
Zaborin et al. (6) 2014	Longitudinal cohort	~50 surgical ICU	16S rRNA	Extreme dysbiosis—single-taxon dominance (Enterococcus, Staphylococcus, Enterobacteriaceae); monoculture state within days	Preceded infectious complications
Schlechte et al. (7) 2023	Prospective + CyTOF immune profiling	51 ICU	16S + mass cytometry	Microbiota-immune metasystem: Enterobacteriaceae enrichment + immature neutrophil phenotype	OR 6.8 (95% CI 1.7–25.3) for nosocomial infection, independent of SOFA
Haak et al. (8) 2021	Prospective + MOFA multi-omics	33 ICU + 13 controls	16S + ITS1 + virome + NMR	Transkingdom integration; SCFAs negatively correlated with fungal abundance; cross-kingdom covariation	Factors 1 and 3 associated with disease state and antibiotic exposure
Salameh et al. (9) 2023	Prospective single-centre	52 ICU	16S (full V)	MMI (Anaerococcus + Enterobacteriaceae vs Parasutterella + Campylobacter) predicts mortality; single-centre derivation	HR 2.5 (95% CI 1.4–4.7) independent of APACHE II; external validation pending

A critical methodological caveat applies throughout this review: the healthy-microbiota reference commonly used to evaluate ICU dysbiosis is an imperfect comparator. Reference datasets predominantly originate from Western, non-ICU cohorts (12, 13), and the pre-admission microbiota of typical ICU patients—often already altered by chronic diseases, medications, and prior antibiotic use—is rarely characterized. The relevant comparator is not an idealized healthy adult microbiome, but the patient’s likely pre-illness ecological and functional state. Dysbiosis severity scores calibrated against healthy controls may therefore systematically misclassify ICU patients whose pre-morbid microbiomes were already disrupted. This caveat should be borne in mind when interpreting all evidence presented below.

To date, no microbiome biomarker has been prospectively validated, and no microbiome-directed intervention has shown Phase III evidence in the general ICU population. Notably, approximately 20% of ICU patients retain relatively preserved microbial diversity during their stay (5). This resilient minority challenges deterministic views of ICU dysbiosis and may provide crucial insights for future prevention strategies.

Central Thesis. We propose that ICU dysbiosis should be viewed not as a collection of isolated taxonomic anomalies but as a putative predictable ecological collapse caused by convergent selective pressures—luminal oxygen enrichment, nitrate availability, and antibiotic-mediated niche clearance. This perspective shifts the focus from individual bacterial taxa to the overall ecosystem’s function, resilience, and restoration. It reframes the therapeutic question from ‘which bacteria should we add?’ to ‘which ecological conditions can we restore, and how quickly?’

This review focuses on five themes that previous literature has insufficiently explored: an ecological framework for ICU dysbiosis highlighting microbial resilience; the host–microbiome adaptive system as a bidirectional, self-amplifying circuit; the debate on whether function or taxonomy should be the therapeutic target; structural reasons for clinical trial failures and strategies for redesign; and a clinically actionable framework based on rapid ecological risk assessment, endotype classification, and targeted intervention selection.

## 2 The healthy Gut microbiota: reference, function, and ICU baseline limitations

### 2.1 Composition and spatial organization

The healthy adult gut harbors four dominant bacterial phyla—Firmicutes, Bacteroidetes, Actinobacteria, and Proteobacteria—with composition varying substantially across individuals, cohorts, diet, geography, and age (12, 13). Rather than fixed numerical benchmarks, what matters clinically is the functional repertoire these communities sustain: obligate anaerobes (Ruminococcaceae, Lachnospiraceae, Clostridiaceae) are the keystone producers of butyrate, propionate, acetate, secondary bile acids, and indole derivatives that sustain colonocyte energy, tight-junction integrity, and immune homeostasis (14, 15). Facultative anaerobes such as Enterobacteriaceae and Enterococcus normally constitute a minor fraction of the colonic community; their dominance in critical illness marks a phase transition—ecosystem collapse with fundamentally altered functional properties—not incremental compositional drift.

Spatial organization is as important as composition. The mucosa-adherent community that directly interfaces with epithelial and immune cells in the lamina propria differs substantially from the luminal community sampled by rectal swabs or fecal specimens. This distinction has direct relevance to critical illness, where barrier failure and immune dysregulation depend primarily on microbial–host interactions at the mucosal surface. Most ICU microbiome studies capture only the luminal community, leaving the mechanistically important mucosa-adherent population largely unmeasured.

### 2.2 Functional homeostasis

#### 2.2.1 Short-chain fatty acids

Microbial fermentation of dietary fiber generates three main short-chain fatty acids (SCFAs). Butyrate supplies 60–70% of the oxidative energy for colonocytes and enhances tight-junction proteins through AMP-activated protein kinase (AMPK) (20, 21). Propionate and acetate enter the portal circulation, influencing hepatic gluconeogenesis and systemic immune response (22). In ICU patients, depletion of SCFA-producing obligate anaerobes substantially reduces these concentrations, compromising colonocyte metabolism, epithelial barrier integrity, and mucosal immune function (8)—effects that are mechanistically more relevant to critical care than resting healthy-state values per se.

#### 2.2.2 Bile acid metabolism and the FXR–TGR5 axis

Gut microbes deconjugate primary bile acids using bile salt hydrolases. Specific taxa, such as *Clostridium scindens* and members of the Lachnospiraceae family, further convert these deconjugated acids into secondary bile acids through 7α-dehydroxylation, activating the receptors FXR and TGR5, influencing lipid metabolism, glucose homeostasis, and mucosal immunity (23, 24). In ICU-associated dysbiosis, loss of these beneficial microbes shifts the bile acid pool towards primary acids, impairing FXR–TGR5 signaling and contributing to parenteral nutrition–associated liver disease (PNALD) (25).

#### 2.2.3 Colonization resistance: an emergent ecological property

Colonization resistance emerges from a fully functioning commensal ecosystem through three complementary mechanisms: competitive nutrient consumption depriving pathogens of essential resources; bacteriocins from *Enterococcus faecalis* and *Lactobacillus* species that directly lyse competing bacteria; and induction of host defenses including sIgA, RegIIIγ, and defensins (34, 35). During ICU dysbiosis, erosion of community diversity progressively dismantles each layer of this emergent resistance, creating the ecological vacancy that permits pathogen dominance.

### 2.3 Host–microbiome immune interactions

The microbiota plays a crucial role in modulating the immune system. In germ-free animals, there is a notable reduction in Peyer’s patches, decreased IgA secretion, and improper differentiation of Tregs and Th17 cells (36, 37). Short-chain fatty acids promote the differentiation of colonic Tregs by inhibiting HDACs (38, 39). Polysaccharide A from *Bacteroides fragilis* enhances IL-10 production through TLR2 stimulation (40), while segmented filamentous bacteria are essential for inducing Th17 cells necessary for mucosal defense (41). Bacterial extracellular vesicles (20–300 nm) transport LPS, peptidoglycan, and nucleic acids across the mucus layer, adding a paracrine dimension to host–microbe interactions (42, 43).

### 2.4 Challenges in defining a healthy ICU baseline

The healthy-microbiota reference commonly used to evaluate ICU dysbiosis is inadequate for critical-care populations. First, reference datasets predominantly originate from Western, non-ICU cohorts (12, 13). Second, the pre-admission microbiota of typical ICU patients—often altered by chronic diseases, medications, and prior antibiotic use—is rarely characterized. Third, most ICU studies depend on fecal or rectal-swab sampling, which overlooks the mucosa-adherent community that directly interacts with immune cells. Fourth, twin studies estimate individual-taxon heritability at only 2–8% (44), indicating significant interindividual variation. The relevant comparator is not an idealized healthy adult microbiome but the patient’s likely pre-illness ecological and functional state—a baseline that is rarely captured in current clinical practice or research.

## 3 Ecological principles of ICU dysbiosis

### 3.1 From ecosystem stability to phase transition

**Figure 2:**
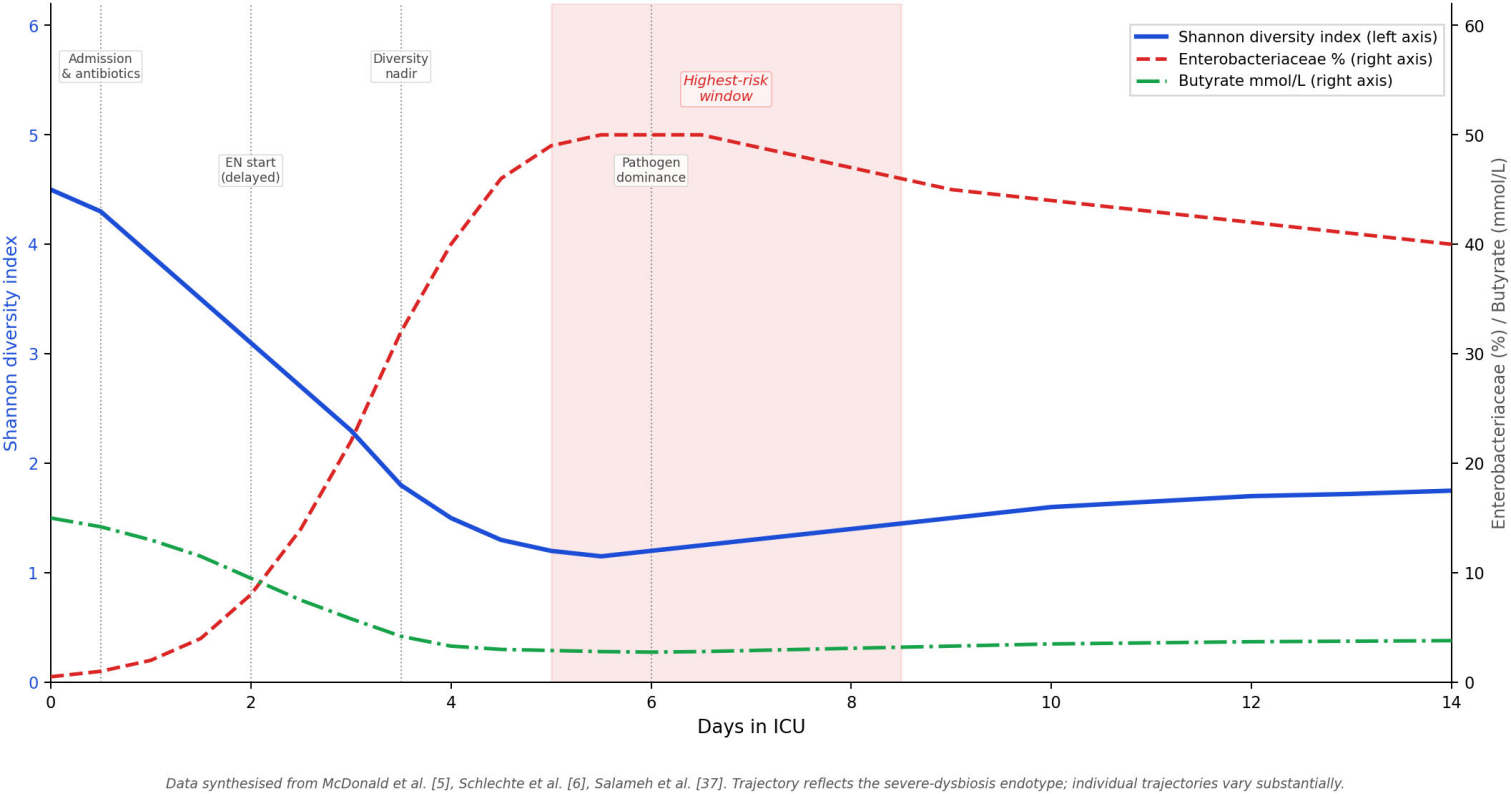
Gut microbiome dynamics during ICU stay (Days 0–14). Shannon diversity index (blue, left axis): falls from ~4.5 at admission to nadir ~1.2 by Day 6–8. Enterobacteriaceae (red dashed, right axis): rises from <1% to ~50% by Day 6. Butyrate (green dash-dot, right axis): declines from ~15 to <3 mmol/L. Highest-risk window (Days 5–8.5) shaded. This figure represents a synthesized trajectory reflecting the severe-dysbiosis endotype across multiple cohorts [McDonald et al. (5), Schlechte et al. (7), Salameh et al. (9)]; trajectories vary substantially by antibiotic exposure and baseline microbiome state. Values are smoothed for illustrative purposes. Approximately 15–20% of patients (resilient subgroup) maintain Shannon diversity >3.0. EN, enteral nutrition.

A healthy gut microbiome demonstrates ecological resilience by absorbing disturbances and returning to its baseline state. This resilience relies on functional redundancy, cross-feeding networks, and niche saturation. ICU conditions simultaneously disrupt these mechanisms, driving what may be characterized as a phase transition—akin to a tipping point in complex systems—shifting from a high-diversity, functional, anaerobe-dominated state to a low-diversity, dysfunctional, pathogen-dominated state (5, 7) (**Figure 2**). Once the system crosses this tipping point, minor interventions such as single-strain probiotics are insufficient for restoration. Ecological concepts introduced in this section underpin the therapeutic framework developed throughout the review.

### 3.2 The three ecological drivers of ICU dysbiosis

ICU dysbiosis is characterized by a significant reduction in microbial diversity and a shift from ecosystems dominated by obligate anaerobes to those dominated by facultative pathogens. This ecological transition is driven by three convergent selective pressures—potentially additive in their effects, with relative contributions varying by patient endotype, antibiotic exposure pattern, inflammatory burden, and timing of intervention:

- Luminal oxygen enrichment. In a healthy colon, luminal oxygen levels are nearly zero—a condition necessary for the dominance of obligate anaerobes (45). During critical illness, mucosal inflammation disrupts epithelial oxygen consumption, allowing oxygen to diffuse into the lumen, disadvantaging obligate anaerobes and favoring facultative or aerotolerant taxa (45).
- Nitrate enrichment from iNOS. Systemic inflammation triggers activation of inducible nitric oxide synthase (iNOS) in the intestinal epithelium, producing luminal nitrate. Enterobacteriaceae possess nitrate reductases enabling them to utilize nitrate as a terminal electron acceptor, providing a metabolic advantage in inflamed gut environments (46).
- Antibiotic-mediated niche clearance. Broad-spectrum antibiotics such as carbapenems and piperacillin-tazobactam eliminate resident microbial communities, creating vacant ecological niches (47, 48). This permits resistant facultative anaerobes to colonize through classical ecological succession—analogous to post-forest-fire succession (49).

The relative dominance of each driver has therapeutic implications: when antibiotic-mediated niche clearance predominates, stewardship and ecological restoration may be prioritized; when inflammation-driven oxygen or nitrate enrichment predominates, strategies targeting the host epithelial environment may be required before microbial repletion can succeed.

### 3.3 Keystone species and functional redundancy

Keystone species are taxa whose removal leads to ecosystem disruption disproportionate to their abundance. In the gut, key anaerobes include *Faecalibacterium prausnitzii* (butyrate production, anti-inflammatory signaling); *Akkermansia muciniphila* (mucus layer maintenance); *Clostridium scindens* (secondary bile acid synthesis, resistance to *Clostridioides difficile* infection [CDI]); and *Lactobacillus* species (barrier support, immune modulation) (14, 50).

The loss of these keystone taxa operates through two distinct mechanisms. First, it eliminates unique functional outputs that no other community member can replicate—for example, 7α-dehydroxylation is performed by a limited set of taxa, so their loss constitutes a direct functional deficit rather than a redundancy problem. Second, it erodes the broader redundancy across shared functions such as butyrate production: when multiple SCFA-producing taxa are simultaneously depleted, the network’s buffering capacity collapses. Restoring a single keystone species may recover a unique function (as with *C. scindens* and secondary bile acid synthesis) but may not restore the broader functional network—which requires multi-taxon ecosystem-level restoration. This provides the conceptual rationale for multi-strain fecal microbiota transplantation (FMT) when community-level redundancy has been severely eroded.

### 3.4 Ecological succession and the therapeutic window

Ecological succession offers a predictive model for ICU dysbiosis trajectories. Pioneer taxa like Enterobacteriaceae and Enterococcus colonize first; without recovery stimuli, they maintain dominance. Keystone anaerobes are the last and least complete to recover (5, 9, 10). This succession pattern highlights a therapeutic window: interventions for community restoration should ideally occur within 24–48 hours of ICU admission, before pioneer taxa establish dominance.

### 3.5 Cross-kingdom ecology

**Figure 3:**
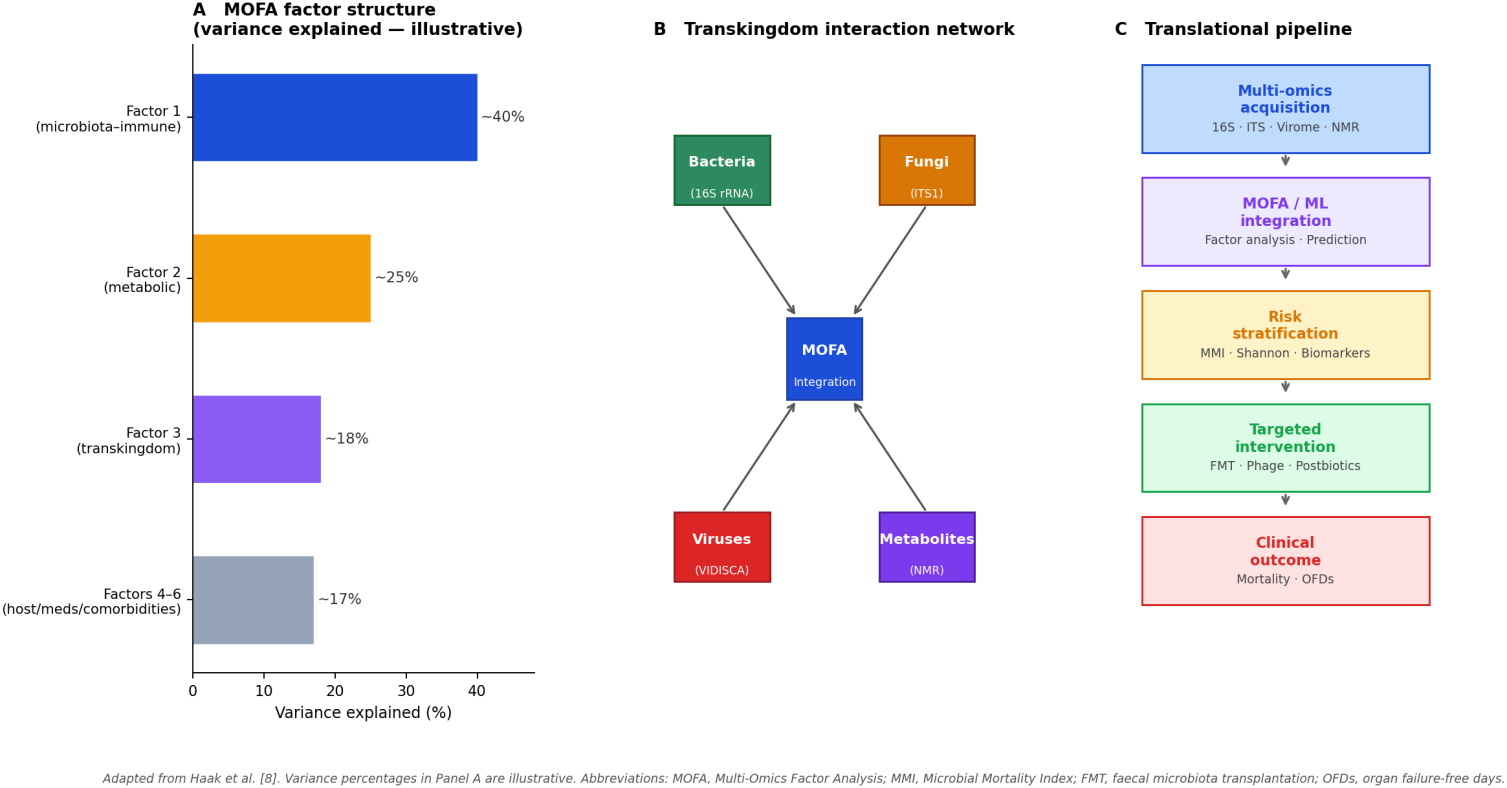
Multi-omics integrative analysis: MOFA factor structure, transkingdom network, and translational pipeline. **(A)** MOFA factor structure (adapted from Haak et al. (8)): Factor 1 (~40% variance)—microbiota–immune axis; Factor 2 (~25%)—metabolic covariation; Factor 3 (~18%)—transkingdom interactions; Factors 4–6 (~17%)—host/medication/comorbidity effects. Variance percentages are illustrative. **(B)** Transkingdom network: bacteria (16S rRNA), fungi (ITS1), viruses (VIDISCA), metabolites (NMR) integrated via MOFA. **(C)** Translational pipeline. MOFA, Multi-Omics Factor Analysis; MMI, Microbial Mortality Index; FMT, fecal microbiota transplantation; OFDs, organ failure-free days.

Haak et al. utilized MOFA (**Figure 3**) to analyze paired 16S, ITS1, VIDISCA-NGS, and NMR data, revealing that bacterial, fungal, viral, and metabolite communities co-vary in ways that bacterial ecology alone cannot explain (8). As Lachnospiraceae and Ruminococcaceae populations decreased, *Candida* and *Aspergillus* expanded—influenced by butyrate deficiency, which weakened epithelial antifungal defenses and promoted fungal adhesion.

Functionally, mycobiome and virome changes during ICU dysbiosis may act through three distinct mechanisms: (i) as markers of bacterial ecosystem collapse, where fungal overgrowth reflects loss of bacterial competitive exclusion; (ii) as amplifiers of barrier injury and immune dysregulation, where *Candida* adhesion and biofilm formation on a compromised epithelium exacerbates mucosal damage; and (iii) as contributors to pathogen fitness, where phage-mediated horizontal gene transfer can alter the competitive landscape among bacterial pathogens. Disentangling these functional roles is a research priority.

### 3.6 Microbial resilience: the missing half of the story

A comprehensive theory of ICU dysbiosis must explain why most patients experience microbiome collapse, while approximately 15–20% do not (5, 9). This resilient subgroup—patients maintaining a Shannon diversity index greater than 3.0 during ICU stays—represents a crucial yet underexplored area. Several mechanisms have been proposed as hypotheses, though direct evidence in human ICU populations is limited: (1) preadmission microbiomes enriched in taxa with broad metabolic versatility; (2) retention of keystone SCFA producers like *F. prausnitzii* or *Roseburia intestinalis*; (3) use of narrow-spectrum antibiotics that spare anaerobes; and (4) host genetic variation in innate immune receptors (TLR4, NOD2) and mucosal barrier genes (44, 47, 48). These remain candidate explanations requiring prospective testing. Future microbiome medicine may depend as much on understanding and fostering resilience as on treating established dysbiosis.

## 4 Methodological challenges in ICU microbiome research

### 4.1 Sequencing technology: 16S vs shotgun vs functional metagenomics

Different sequencing approaches answer different questions, and the choice of method should be driven by the biological question rather than cost alone. 16S rRNA amplicon sequencing is suited to characterizing broad taxonomic community structure, but the choice of variable region affects taxon detection, generating batch effects that complicate cross-study comparisons (51). Additionally, 16S sequencing cannot differentiate between live and dead organisms, identify functional gene content, or provide absolute abundances, resulting in compositional data artefacts (52).

Shotgun metagenomics provides species-level resolution and functional gene content, suited to questions about genetic potential and strain-level diversity, though it costs substantially more and host DNA contamination from damaged epithelial cells can constitute over 90% of sequences in ICU patients. Functional metagenomics—encompassing metatranscriptomics (active gene expression), metaproteomics (expressed proteins), and metabolomics (realized functional outputs)—is the most appropriate method for answering function-first questions, including SCFA concentrations, bile acid profiles, and indole pathway activity. These approaches have been notably underutilized in ICU cohort studies.

### 4.2 Sampling limitations

Most ICU studies rely on rectal swabs instead of whole-stool samples. Whole-stool sampling may improve representation of fecal community structure and metabolite profiles, but it does not resolve the core mechanistic sampling problem: both approaches primarily reflect the luminal compartment, not the mucosa-adherent community that directly interacts with immune cells in the lamina propria (52). Since mucosal biopsies are not clinically feasible, a significant gap persists between measurable data and the mechanistically important microbial communities.

### 4.3 Compositional data artefacts and absolute quantification

Standard 16S sequencing generates compositional data, where a change in one taxon inevitably causes apparent reciprocal changes in others (52). The reported ‘expansion’ of Enterobacteriaceae in ICU dysbiosis might indicate actual growth or could be a mathematical artifact. Absolute quantification methods (spike-in controls, flow cytometry, quantitative PCR) can clarify this ambiguity but are rarely used.

### 4.4 Contamination, batch effects, and reproducibility

Low-biomass samples are particularly vulnerable to reagent contamination (*Pseudomonas*, *Ralstonia*, *Burkholderia*) (53). Published audits suggest that systematic negative controls are frequently omitted in ICU microbiome studies. Batch effects from varying sequencing runs and bioinformatic pipelines generate study-specific compositional signatures, complicating meta-analysis.

### 4.5 Causal inference from observational data

Most evidence connecting dysbiosis to clinical outcomes originates from observational cohorts, where illness severity acts as a significant confounder. Mendelian randomization has not yet been applied to ICU dysbiosis (52). Until causal methods are employed, claims linking dysbiosis to outcomes should be regarded as associations rather than established pathophysiology.

## 5 Mechanisms linking dysbiosis to organ dysfunction

This section examines mechanistic pathways through which gut dysbiosis contributes to systemic pathology, proceeding from most proximate to most distal: (1) anaerobe loss → SCFA depletion → colonocyte metabolic failure → epithelial barrier disruption; (2) immune and metabolic dysregulation, simultaneously and bidirectionally coupled; and (3) organ-specific gut–organ axes with explicitly graded evidence quality. Human ICU evidence is distinguished from animal model or *in vitro* evidence throughout.

### 5.1 Intestinal barrier failure

The intestinal barrier relies on a steady supply of SCFAs (54, 55). When butyrate-producing commensals are depleted, colonocytes shift to glutamine oxidation, resulting in reduced ATP production and downregulation of occludin, claudin-1, and ZO-1 (21, 56). Butyrate activates AMPK, promoting proper membrane localization of tight-junction proteins (20). Simultaneously, the depletion of *Akkermansia muciniphila* thins the mucus layer, and LPS from expanded Enterobacteriaceae signals through TLR4 to upregulate myosin light chain kinase, increasing paracellular permeability (54). This sequence—anaerobe loss → SCFA depletion → colonocyte metabolic failure → tight-junction downregulation → mucus thinning → microbial product translocation—constitutes the core ICU-associated barrier failure cascade.

Superimposed on this core sequence, pathogen-specific amplifiers can accelerate barrier failure. *Clostridioides difficile* toxins A and B glucosylate Rho GTPases (57), dramatically worsening paracellular integrity in the subset of patients who develop CDI. The primary cell-death pathway responsible for human intestinal barrier failure during critical illness remains unidentified, representing an important knowledge gap.

### 5.2 Immune dysregulation: the microbiota–immune metasystem

Schlechte et al.’s concept of a ‘microbiota-immune metasystem’ highlights that dysbiosis and immune dysfunction occur simultaneously as failures within a unified system (7). During Enterobacteriaceae expansion, circulating neutrophils transitioned from mature effector phenotypes to immature progenitors, increasing the risk of nosocomial infections (OR 6.8, 95% CI 1.7–25.3), independent of SOFA scores (7). Additionally, SCFA depletion impairs colonic Treg differentiation (38, 39), while bile acid pool disruption affects mucosal IgA class switching.

### 5.3 The host–microbiome adaptive system: a bidirectional circuit

Recent data suggest a bidirectional interaction functioning as a coupled dynamical system rather than a straightforward cause-and-effect sequence. Host inflammation increases luminal oxygen and nitrate levels and triggers release of antimicrobial peptides, selectively eliminating obligate anaerobes. This leads to SCFA depletion, barrier failure, and pathogen proliferation. Pathogen growth facilitates systemic translocation of LPS and other microbial products, intensifying systemic inflammation—completing the self-amplifying circuit. Once a positive feedback loop surpasses a threshold, it can sustain itself even after the initial trigger has resolved, explaining why dysbiosis can persist long after clinical recovery.

### 5.4 Metabolic failure beyond SCFAs

Tryptophan–indole axis: *Lactobacillus* converts tryptophan into indole derivatives supporting intestinal homeostasis through the aryl hydrocarbon receptor (AhR) and preserving GLP-1 secretion. Wang et al. demonstrated that total parenteral nutrition (PN) decreased *Lactobacillus* levels, depleted indole derivatives, and suppressed GLP-1 via AhR, linking PN-induced dysbiosis to impaired glucose regulation (33).

Bile acid cascade: The loss of 7α-dehydroxylating commensals shifts the bile acid pool toward primary bile acids, impairing FXR–TGR5 signaling, reducing FGF19 levels, and disrupting hepatic bile acid homeostasis. In PN-dependent patients, these changes contribute to cholestasis and PNALD (25).

### 5.5 Gut–organ axes: a critical appraisal of evidence quality

Gut–organ signaling functions through four distinct axes, presented in order of decreasing strength of human ICU evidence.

#### 5.5.1 Gut–liver axis (strongest human ICU evidence)

The portal circulation carries gut-derived microbial products directly to the liver, where Kupffer cells detect them through TLR4, TLR2, TLR5, NOD1, and NOD2 (58). The liver normally clears more than 95% of portal endotoxin; when the intestinal barrier fails, microbial products enter the systemic circulation. This axis is well-characterized in human critical illness, supported by direct portal LPS measurements and consistent correlations between dysbiosis severity and liver dysfunction markers.

#### 5.5.2 Gut–lung axis (moderate evidence; metabolite-mediated stronger than viable translocation)

Dickson et al. found enrichment of gut-derived Bacteroides in the lower respiratory tract microbiome of ARDS patients (59, 60). Metabolite-mediated signaling via SCFAs reaching the pulmonary vasculature is evidenced by *in vitro* and murine studies (61). Evidence for viable bacterial translocation is weaker.

#### 5.5.3 Gut–kidney axis (moderate evidence; primarily animal/observational human data)

SCFAs interact with GPR41 and GPR43 on renal tubular cells, influencing renin secretion (62). Urease-producing gut bacteria produce uremic toxins (p-cresol, indoxyl sulfate) that accumulate during AKI and contribute to systemic immune paralysis (62).

#### 5.5.4 Gut–brain axis (weakest direct ICU evidence)

The gut–brain axis operates via vagal afferent signaling, HPA axis regulation by microbial metabolites, and immune-driven neuroinflammation (63). A reduction in SCFAs decreases vagal tone and has been suggested as a factor in ICU-associated delirium. Direct human ICU evidence remains limited.

## 6 Clinical consequences of dysbiosis

### 6.1 Sepsis

Sepsis—life-threatening organ dysfunction resulting from a dysregulated host response to infection (64)—represents the most direct clinical translation of the ecological-collapse thesis: when gut barrier failure permits translocation of LPS, peptidoglycan, and microbial DNA into the portal and systemic circulation, it intensifies the cytokine cascade of septic shock (3, 4)—a process mechanistically downstream of SCFA depletion and tight-junction failure. Dysbiosis severity at ICU admission is independently linked to subsequent septic complications, even after adjusting for SOFA score (7, 9).

### 6.2 Nosocomial infection

Progressive enrichment of Enterobacteriaceae predicts nosocomial infections (OR 6.8, 95% CI 1.7–25.3, independent of SOFA score) (7)—the direct clinical consequence of colonization resistance failure. Intestinal overgrowth often precedes bloodstream infections and ventilator-associated pneumonia (65, 66). FMT, with a cure rate exceeding 90% for recurrent CDI (29, 30), demonstrates that microbiome restoration is clinically feasible and effective when the ecological target is clearly defined.

### 6.3 Acute gastrointestinal injury

ESPEN defines intestinal failure as insufficient GI function to absorb macronutrients without intravenous supplementation (67). The AGI grading system (I–IV) has been validated in multiple cohorts; AGI grades II or higher occur in 30–60% of ICU patients and independently predict 28-day and 90-day mortality (68, 69). The trajectory of AGI closely mirrors microbiome collapse—both follow a similar temporal course driven by the same three ecological drivers—suggesting common pathophysiology.

### 6.4 Multiple organ dysfunction syndrome

MODS can be conceptualized as systemic ecosystem collapse: the gut fails to contain its microbial ecology, leakage of microbial products triggers a self-amplifying inflammatory cycle that propagates to distal organs via the four gut–organ axes. In CLP models, germ-free mice did not exhibit the same MODS pattern (3, 4, 70). In humans, systemic LPS levels correlate with the severity of organ failure. Direct causal evidence from restoration trials has not yet been established.

### 6.5 Mortality

Shannon diversity and pathogen burden independently predict 28-day mortality across different cohorts (5, 7, 9). The Microbial Mortality Index (MMI)—contrasting Anaerococcus and Enterobacteriaceae (higher mortality) with Parasutterella and Campylobacter (lower mortality)—predicted mortality beyond APACHE II score (HR 2.5, 95% CI 1.4–4.7) in the derivation cohort (9). These associations are consistent with the ecological-collapse thesis but should currently guide research stratification design rather than clinical practice, as these biomarkers await prospective external validation.

## 7 Is dysbiosis the wrong target? The function versus taxonomy debate

For the purposes of this section, ‘ecosystem function’ refers to the set of community-level outputs that directly modulate host physiology: SCFA production and colonocyte energetics; bile acid transformation capacity; tryptophan–indole pathway activity; colonization resistance; epithelial barrier support; and immune modulation through metabolite-pattern recognition receptor interactions. These functional outputs ultimately determine the clinical relevance of the gut microbiome, regardless of which specific taxa generate them.

Current research primarily focuses on restoring microbial composition based on taxonomic identity. However, growing evidence questions whether composition is the main determinant of clinical outcomes. Two patients may have entirely different microbial compositions yet produce similar amounts of butyrate, show comparable colonization resistance, and generate similar immune signals. Conversely, patients with similar Shannon diversity might exhibit vastly different functional states if one retains butyrate-producing keystone species and the other does not.

This function-first perspective is supported by empirical evidence. Buffie et al. demonstrated that restoring a single keystone species, *C. scindens*, was sufficient to recover secondary bile acid production and resistance to CDI, without restoring overall diversity [Buffie CG, Bucci V, Stein RR, et al. *Nature* 2015; 517:205–208]. Similarly, Wang et al. found that restoring the indole pathway—rather than merely increasing *Lactobacillus* abundance—was the functionally relevant outcome for PN-induced dysbiosis (33).

Implications for biomarker development: A functional biomarker panel directly measuring SCFA concentrations, bile acid profiles, and indole derivatives offers a more accurate reflection of the clinically relevant ecosystem state than a taxonomic diversity index, can be obtained within hours, and interpreted without bioinformatic expertise. This represents a high-priority research direction in biomarker development.

Implications for intervention design: Targeting function over taxonomy makes postbiotic strategies—directly supplementing functional outputs—more ecologically rational as first-line options. The taxonomy-versus-function debate reframes the research hierarchy: prioritize measuring function, use taxonomy to explain it, and directly intervene on function when community restoration is not feasible.

## 8 Biomarkers and predictive models

### 8.1 Protein biomarkers of barrier injury

*Intended clinical use: diagnosis of epithelial barrier injury and early risk stratification* (**Table 2**).

**Table 2:** Biomarkers of Intestinal Barrier Injury and Microecological Disruption.

Biomarker	Biological basis	Se/Sp	AUC	Availability	Grade	Key limitation
I-FABP (16)	Cytosolic enterocyte protein; released before histological necrosis	Se 80–90% Sp 85–95%	0.85–0.95	ELISA; 2–4 h	High (ESPEN 2025)	Cutoff 87–355 pg/mL; no impact study
Citrulline (17, 18)	Synthesised exclusively by functional enterocytes; correlates with enterocyte mass	Se 70–85% Sp 75–88%	0.75–0.88	HPLC/LC-MS	Moderate–High	AKI confound (~30% ICU); cannot distinguish reduced mass from reduced clearance
D-Lactate (19)	Bacterial fermentation product reflecting barrier permeability	Se 65–80% Sp 70–85%	0.70–0.82	Enzymatic assay	Moderate	Non-specific; renal confound; adjunct marker only
Shannon Index (5, 9)	Composite microbial diversity measure (richness + evenness)	Continuous	0.75–0.91	16S; 24–72 h	Moderate–High	Incompatible with ICU decision timeframes; relative abundance artefact risk
MMI (9)	4-taxon ratio (Anaerococcus + Enterobacteriaceae vs Parasutterella + Campylobacter)	HR metric	0.80–0.85 (derivation)	qPCR potential; not validated	Moderate (no external validation)	Single-centre derivation (n=52); no external validation; AUC unstable

I-FABP: A 15-kDa protein specific to enterocytes, released before histological necrosis. Piton et al. demonstrated elevated I-FABP levels independently predicted 28-day mortality (AUCs 0.85–0.95) (16). ESICM guidelines acknowledge I-FABP as a marker of acute enterocyte injury (71). A significant limitation is the wide variation in cutoff values (87–355 pg/mL), and no impact study has shown that I-FABP measurement influences clinical decisions.

Citrulline: Synthesized exclusively by functional enterocytes; steady-state plasma concentration correlates with enterocyte mass (AUC 0.75–0.88) (17, 18). However, acute kidney injury, affecting ~30% of ICU patients, reduces renal citrulline clearance, complicating interpretation.

D-Lactate: A bacterial fermentation product indicating barrier permeability (AUC 0.70–0.82) (19). Non-specific and influenced by renal function; useful only as an adjunctive measure.

### 8.2 Microbiome-based biomarkers

*Intended clinical use: dysbiosis-risk stratification, endotype classification, and outcome prediction.*

Shannon diversity (AUC 0.75–0.91) consistently serves as the most reliable microbiome predictor (5, 9). The MMI simplifies this into a four-taxon ratio but remains in development. A major challenge is the 24–72 hour timeframe required for 16S sequencing. Rapid qPCR panels targeting 5–10 key dysbiosis taxa could reduce turnaround to 2–4 hours but have not been validated. Whether microbiome-derived risk scores can outperform SOFA in predicting organ failure–free days remains an unanswered question; no prospective head-to-head comparison exists.

### 8.3 Machine-learning predictive models

*Intended clinical use: outcome prediction and endotype classification — currently development-stage tools only.*

All published critical-care microbiome machine learning models—including MMI (9), the XGBoost model for antibiotic-associated diarrhea (Cui et al.) (72), and the EN-associated diarrhea model (Liao et al.) (73)—remain solely at the derivation stage. To be clinically useful, a model must undergo external validation in a prospective, pre-registered independent cohort with pre-specified calibration, net-benefit analysis, and health-economic comparison against SOFA. None of the models in this field have completed these essential steps.

## 9 Microbiome-directed therapeutic strategies

### 9.1 Early enteral nutrition (GRADE: HIGH)

The evidence for each therapeutic strategy is summarized in **Table 3**. Early enteral nutrition (EN), initiated within 24–48 hours at trophic rates of 10–20 mL/h, is the most evidence-based strategy for supporting the gut microbiome. Clinical guidelines from the Society of Critical Care Medicine/American Society for Parenteral and Enteral Nutrition (SCCM/ASPEN) and European Society of Intensive Care Medicine (ESICM) endorse this method (26, 27). EN preserves villous architecture, enhances tight-junction protein expression, supports secretory IgA and GALT function, and maintains SCFA-producing commensals (74, 75). Feeding intolerance affects 30–60% of patients (76). Patients with AGI grades I–II can tolerate cautious EN combined with prokinetics, while those with AGI grades III–IV require EN suspension (71, 77, 78).

**Table 3:** Microbiome-Directed Therapeutic Strategies: GRADE Evidence Matrix.

Strategy	Mechanism	Grade	Key evidence	Primary limitations	Recommendation
Early Enteral Nutrition	Fermentable substrate → SCFA production; villous maintenance; GALT support	HIGH	SCCM/ASPEN 2016; ESICM 2017; multiple RCTs (26, 27)	Intolerance 30–60%; AGI Grade III–IV requires pause	Strong: initiate within 24–48 h
Antimicrobial Stewardship	Limits antibiotic-mediated niche clearance; preserves anaerobe community	MODERATE–HIGH	IDSA/SHEA guidelines; PCT-guided RCTs (28)	De-escalation challenging in early sepsis	Strong: apply universally
FMT	Ecosystem-level restoration; colonization resistance	HIGH (rCDI) LOW (general ICU)	>90% rCDI cure (29, 30); no Phase II/III for non-rCDI ICU	MDR transmission (85); no standardized protocol	rCDI: strong. General ICU: research only
Probiotics/Synbiotics	Single-strain competitive inhibition (ecologically misaligned)	LOW–MODERATE	Largest RCT n=2,653: no VAP/mortality benefit (31)	Ecological misalignment; absent stratification; biological efficacy limited	NOT recommended for routine use
Phage Therapy	Targeted lysis of CRE/VRE; spares commensals	VERY LOW	Preclinical; Phase I/II ongoing (32)	Immunogenicity; phage resistance; regulatory pathway unclear	Clinical trials only
Postbiotics/SCFA Suppl.	Direct SCFA supply; tryptophan-indole-AhR pathway (33)	VERY LOW	Preclinical; PN-indole mechanism defined (33)	Colonic delivery challenge; off-target effects possible	Research stage only

### 9.2 Antimicrobial stewardship (GRADE: MODERATE–HIGH)

Antimicrobial stewardship is among the most cost-effective strategies for safeguarding the microbiome, targeting Driver 3—antibiotic-mediated niche clearance (79, 80). Five key practices maximize microbiome protection: restricting carbapenems and piperacillin–tazobactam; promptly de-escalating therapy; utilizing procalcitonin levels to guide duration (28); opting for cefepime over carbapenems when isolates are susceptible; and obtaining appropriate cultures before initiating carbapenem treatment.

### 9.3 Probiotics and synbiotics: why trials have failed and what comes next

The largest RCT (n=2,653) found that *Lactobacillus rhamnosus* GG did not reduce VAP or mortality (31). Meta-analyses confirm no mortality impact (81, 82). Understanding the reasons behind these null results is more instructive than the findings themselves:

- Ecological misalignment: Probiotic strains cannot colonize a colon where 55–80% of the microbiota consists of Enterobacteriaceae without first restoring the niche.
- Absent stratification: No completed trial selected patients based on documented dysbiosis.
- Wrong timing: Many trials began probiotics after the diversity nadir (Day 3–5); preemptive deployment within 12–24 hours remains untested.
- Soft endpoints: VAP and ICU diarrhea are insufficient; mortality and organ failure-free days are necessary.
- Single-strain design: A single strain cannot substitute for the functional redundancy of hundreds of anaerobic species.

An equally important alternative interpretation is that single-strain, non-colonizing probiotic strains may have genuinely limited biological efficacy in the context of severe ICU dysbiosis—not merely insufficient trial power. The two explanations are not mutually exclusive. Until stratified trials with mortality endpoints are completed, this ambiguity cannot be resolved. Reframing: stratified, preemptive, multi-strain trials in confirmed dysbiotic patients with mortality as the primary endpoint have yet to be conducted.

### 9.4 Fecal microbiota transplantation (GRADE: HIGH for rCDI; LOW for general ICU)

FMT has demonstrated a cure rate exceeding 90% for recurrent CDI (29, 30, 83, 84), serving as compelling evidence for microbiome restoration in clinical settings. Applying FMT in general ICU settings presents unique challenges: immune dysfunction increases engraftment failure risk; severe barrier injuries increase translocation potential; and MDR organism transmission is a documented risk (85). Safety-monitored Phase II/III RCTs are essential for non-rCDI ICU applications.

### 9.5 Parenteral nutrition: microbiome mechanism

When EN is not possible, PN sustains life but often leads to dysbiosis. Wang et al. found that PN reduced *Lactobacillus* levels, depleted tryptophan–indole derivatives, and suppressed GLP-1 through AhR (33). Multi-component lipid emulsions have the most substantial evidence in preventing PNALD (86).

### 9.6 Emerging strategies (GRADE: VERY LOW)

Postbiotics/SCFA supplementation (tributyrin, sodium butyrate): mechanistically rational, with challenges in attaining adequate colonic concentrations.

Phage therapy: targeted CRE/VRE lysis without collateral damage to commensal bacteria (32), with Phase I/II trials ongoing. Challenges include immunogenicity, phage resistance emergence, and undefined PK/PD in ICU settings.

Engineered bacteria: synthetic-biology approaches to produce specific metabolites or deliver targeted antimicrobials remain at the preclinical stage (87).

GLP-2 analogues and FXR agonists: Teduglutide enhances epithelial proliferation in short bowel syndrome and intestinal failure (88); this mechanism is biologically plausible but not yet validated for ICU microbiome restoration or barrier repair. FXR agonists (obeticholic acid) address bile acid dysmetabolism (89); evidence for PNALD specifically is extrapolated from other hepatic conditions. ICU-specific RCTs are necessary for both.

## 10 Why have clinical trials failed? A systematic analysis

### 10.1 Patient heterogeneity: the stratification problem

ICU patients exhibit significant variability in baseline microbial composition, illness severity, antibiotic exposure, and nutritional status. Including patients with preserved microbial diversity (Shannon >3.0; ~20% of ICU patients) alongside those with nearly complete anaerobe collapse likely hinders detection of treatment effects.

### 10.2 Ecological misalignment

Most interventions prioritized biological plausibility over ecological compatibility. Introducing *Lactobacillus rhamnosus* GG into an Enterobacteriaceae-dominated community was ecologically analogous to planting a single tree species in an active forest fire.

### 10.3 The therapeutic window has been missed

Interventions to prevent diversity collapse should ideally be implemented within 12–24 hours of ICU admission—a proposed therapeutic window derived from ecological succession models, not yet empirically validated in human ICU trials. Most completed trials began interventions after Day 1, typically on Days 2–3, which was often too late given observed succession dynamics.

### 10.4 Endpoint selection

VAP and ICU-related diarrhea are important surrogate outcomes but do not directly reflect patient-centered endpoints. Mortality and organ failure–free days have seldom been primary endpoints in microbiome trials.

### 10.5 Infrastructure deficit

ICU clinical decisions require minutes to hours; 16S sequencing takes 24–72 hours. Rapid qPCR panels can reduce this to 2–4 hours but have not been validated. Sequencing costs ($200–$1,000 per sample) and the need for specialized bioinformatics further hinder practical implementation.

## 11 Current controversies

### Controversy 1: Is dysbiosis a cause or consequence of organ dysfunction?

For causality: (a) Germ-free animals did not develop MODS in CLP models; (b) transplanting ICU patient microbiota into germ-free recipients led to multi-organ injury; (c) dysbiosis occurred before nosocomial infections in longitudinal cohorts (7); (d) the microbiota–immune metasystem link remained significant after SOFA adjustment (7).

Against direct causality: (a) Illness severity confounds associations; (b) murine CLP models fundamentally differ from the human ICU environment; (c) no restoration trial has demonstrated improved hard clinical outcomes.

Resolution: Only microbiome-restoration trials demonstrating improved survival can definitively settle this debate. Mendelian randomization in biobank-linked ICU cohorts represents a complementary approach (52). Until such evidence is available, dysbiosis remains a consistent correlate and plausible amplifier of organ dysfunction—not a proven independent cause.

### Controversy 2: Can microbiome markers outperform SOFA?

Shannon diversity and MMI offered prognostic insights surpassing APACHE II/SOFA in derivation cohorts, and MOFA factors identified cross-kingdom covariation that clinical scores failed to detect. However, these measures have not undergone external validation, turnaround times are too long for current clinical use, and no health-economic analysis exists. These measures complement established clinical severity scores rather than replace them.

## 12 Knowledge gaps and future directions

### 12.1 Five priority knowledge gaps for the next five years

- Gap 1 — Defining a Healthy ICU Microbiome: Large-scale pre-admission sampling in elective surgical and high-risk outpatient populations is essential.
- Gap 2 — Resilience Mechanisms: Prospective genomic, microbiome, and metabolic analyses comparing resilient and non-resilient patients under similar conditions.
- Gap 3 — Functional Biomarkers: Can microbial functional outputs (SCFAs, secondary bile acids, indole derivatives) serve as bedside biomarkers for endotype classification?
- Gap 4 — Microbiome vs SOFA: No prospective head-to-head comparison with pre-specified decision algorithms and health-economic net benefit analysis exists.
- Gap 5 — Precision Ecological Interventions: Stratified trials with confirmed dysbiosis, endotype-matched intervention, and mortality as the primary endpoint.

### 12.2 Translational roadmap 2026–2036

**Figure 4:**
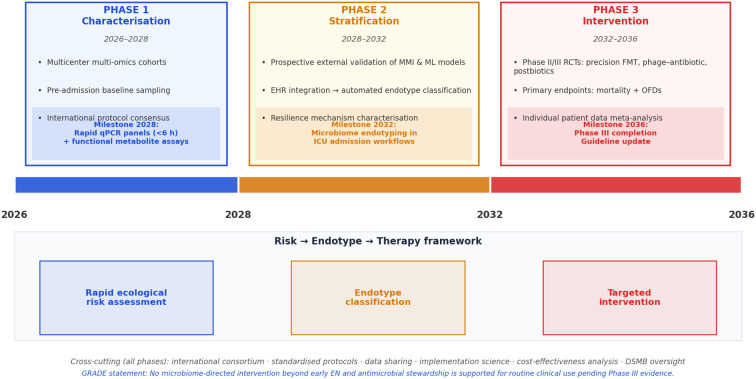
From dysbiosis to intervention: a translational roadmap (2026–2036). Phase 1 Characterization (2026–2028, blue): multicenter multi-omics cohorts, pre-admission baseline sampling, protocol standardization. Milestone 2028: rapid qPCR panels (<6 h turnaround) and functional metabolite assays. Phase 2 Stratification (2028–2032, amber): external model validation, EHR integration, endotype-based eligibility criteria. Milestone 2032: endotyping in ICU admission workflows. Phase 3 Intervention (2032–2036, red): Phase II/III RCTs of precision FMT, phage-antibiotic combinations, postbiotics; primary endpoint mortality and organ failure-free days. Milestone 2036: Phase III completion, practice guidelines updated, Risk–Endotype–Therapy framework validated. Cross-cutting requirements (all phases): international consortium, standardized protocols, data sharing, implementation research, cost-effectiveness analysis, DSMB oversight.

The translational roadmap is illustrated in **Figure 4**. Phase 1—Characterization (2026–2028): Standardized multi-omics sampling within multicenter prospective cohorts; pre-admission baseline studies; international protocol consensus. *Milestone 2028:* valdated rapid qPCR and metabolite dysbiosis panels (<6 h turnaround).

Phase 2—Stratification (2028–2032): Prospective external validation of MMI and ML models; EHR integration for automated endotype classification; resilience mechanism characterization. *Milestone 2032:* microbiome endotyping embedded within ICU admission workflows.

Phase 3—Intervention (2032–2036): Endotype-stratified Phase II/III RCTs focusing on precision FMT consortia, phage–antibiotic combinations, and postbiotic strategies. Primary endpoints: mortality and organ failure–free days. Individual patient data meta-analysis. *Milestone 2036:* Phase III completion, guideline update, Risk–Endotype–Therapy validated.

## 13 Discussion

Over two decades of gut microbiome research in critical illness have produced compelling biology but limited clinical translation. The gap is structural rather than biological. Mechanistically, the evidence is consistent: ecological disruption is real, reproducible, and strongly associated with adverse outcomes. Oxygen enrichment, nitrate advantage, antibiotic-mediated niche clearance, SCFA depletion, barrier failure, and immune-metasystem dysregulation are well-characterized mechanisms (see Sections 3–5 for full exposition).

The central thesis—that ICU dysbiosis resembles a predictable ecological collapse driven by convergent selective pressures—is falsifiable and has survived observational testing. Consistent patterns emerge across independent cohorts, driven by the same three ecological forces, following a stereotyped succession trajectory. A definable resilient minority (~15–20% of patients) maintains diversity under comparable clinical stress; their biology systematically differs from those who collapse, yet remains largely unstudied. The thesis remains unproven at the interventional level: the strongest test—a trial demonstrating that preventing ecological collapse improves hard outcomes—has not been conducted. Completing that trial is the field’s highest priority.

Three structural failures explain the translational gap. The *diagnostic deficit*: without real-time microbiome data, clinicians cannot stratify patients, identify the therapeutic window, or assess intervention response. The *trial-design deficit*: interventions tested without patient stratification, at the wrong time, with wrong endpoints, and without ecological alignment. The *infrastructure deficit*: no multicenter consortium with standardized protocols exists, rendering meta-analysis impossible and individual trials persistently underpowered.

A clinically actionable platform requires three components. First, rapid ecological risk assessment: point-of-care qPCR panels (target: 2–4 hours), if prospectively validated, could provide admission-time microbial context paired with functional metabolite assays. Second, endotype classification: a validated algorithm translating diagnostic data into four clinical endotypes (preserved ecosystem, early dysbiosis, established dysbiosis, pathogen monoculture). Third, ecologically matched intervention: the endotype determines the treatment; single-strain probiotics are ecologically misaligned with all endotypes beyond preservation.

The Risk → Endotype → Therapy framework is conceptually grounded, with each component supported by published precedents, but none has been prospectively validated as an integrated clinical pathway. Building that evidence—through multicenter consortia, validated rapid diagnostics, and endotype-stratified Phase II/III RCTs with mortality as the primary endpoint—is the critical work of the next decade.

## 14 Conclusion

Gut dysbiosis in critical illness is best conceptualized as a predictable ecological collapse—a phase transition hypothesis from high-diversity, keystone anaerobe-dominated communities to low-diversity, pathogen-dominated monocultures—driven by three converging selective pressures: luminal oxygen enrichment from mucosal inflammation, nitrate provision from iNOS, and antibiotic-mediated niche clearance. This ecological reframing shifts the therapeutic question from which bacteria to supplement to which ecological conditions to restore, and on what timeline.

The host–microbiome adaptive system is a bidirectional, self-amplifying circuit. Once past the ecological tipping point, this circuit sustains itself independently of the original precipitant. Ecosystem-level restoration—not supplementation—is the appropriate intervention, deployed before pioneer species establish ecological dominance. The function-versus-taxonomy debate clarifies that the clinically relevant target is ecosystem function—SCFA production, colonization resistance, bile acid synthesis—not species inventory.

The 15–20% of patients who maintain microbial resilience throughout ICU admission represent a natural biological control. Understanding what protects this minority may yield prevention strategies as important as any therapeutic advance. Future microbiome medicine may depend as much on fostering resilience as on treating established dysbiosis.

Current clinical evidence is consistent but not causal. No restoration trial has demonstrated improved survival. Only early enteral nutrition and antimicrobial stewardship meet the evidence threshold for routine use. The field’s most urgent translational priorities are: prospective validation of rapid functional diagnostic panels; development and external validation of endotype-classification algorithms; establishment of multicenter consortium infrastructure with standardized protocols; and completion of endotype-stratified, pre-emptive RCTs with mortality as the primary endpoint. Achieving these milestones within a Risk → Endotype → Therapy framework is the necessary precondition for precision microbiome medicine to become a clinical reality in the ICU.
